# In Vitro Insights into Bacteriocin-Mediated Modulation of Chicken Cecal Microbiota

**DOI:** 10.3390/ijms26020755

**Published:** 2025-01-17

**Authors:** Amal Mamjoud, Séverine Zirah, Eric Biron, Omar Fliss, Ismail Fliss

**Affiliations:** 1 Communication Molecules and Adaptation of Microorganisms (MCAM), Muséum National d’Histoire Naturelle, Centre National de la Recherche Scientifique, 75005 Paris, France; amal.mamjoud.1@ulaval.ca; 2 Food Science Department, Food and Agriculture Faculty, Université Laval, Quebec, QC G1V 0A6, Canada; omar.fliss.1@ulaval.ca; 3 Institute of Nutrition and Functional Foods, Université Laval, Quebec, QC G1V 0A6, Canada; eric.biron@pha.ulaval.ca; 4 Faculty of Pharmacy, Université Laval, Quebec, QC G1V 0A6, Canada

**Keywords:** antimicrobial peptides, microcins, nisin, pediocin, antibiotics, microbiota, batch fermentation, poultry

## Abstract

Reducing the use of antibiotics in animal husbandry is essential to limit the spread of resistance. A promising alternative to antibiotics resides in bacteriocins, which are antimicrobial peptides produced by bacteria showing a great diversity in terms of spectrum of activity, structure, and mechanism of action. In this study, the effects of diverse bacteriocins on the composition and metabolic activity of chicken cecal microbiota were examined in vitro, in comparison with antibiotics. Different impacts on microbiota composition were revealed by 16S metabarcoding, with colistin having the most dramatic impact on diversity. Bacteriocins produced by Gram-negative bacteria, microcins J25 and E492, did not significantly influence the microbiota composition. In contrast, bacteriocins from Gram-positive bacteria impacted the abundance of lactic acid bacteria, with nisin Z showing the most impact while pediocin PA-1 (M31L) exhibited a moderate effect at the highest concentration tested. This study emphasizes the potential of bacteriocins as alternatives to antibiotics in poultry to protect from pathogens such as *Salmonella*, *Clostridium*, and *Enterococcus*.

## 1. Introduction

For over seventy years, antibiotics have been used in livestock production to treat or prevent bacterial diseases and enhance growth performance [1,2]. The overuse of antibiotics has contributed to the emergence and dissemination of antibiotic resistance, which represents a major concern for animal and human health from a One Health perspective [3,4]. This growing concern has driven regulatory bodies to implement guidelines on antibiotic usage in livestock production [4,5]. However, antibiotic use in animal production is not uniformly distributed, documented, or regulated around the world and thus still contributes significantly to the emergence and dissemination of multidrug-resistant (MDR) bacteria [6,7]. Therefore, alternatives to antibiotics are urgently needed [8,9,10].

Poultry production represents a thriving and rapidly growing industry worldwide, yet it faces important challenges [11]. Poultry flocks can be affected by infections by Gram-negative bacteria such as the enterobacteria *Escherichia coli* and *Salmonella* spp. [12] as well as Gram-positive bacteria such as *Clostridium* [13] and *Enterococcus* spp. [14]. Besides chicken morbidity and mortality and altered poultry production, certain of these bacteria represent a threat for human health as foodborne pathogens [13,15]. In addition, broiler chickens represent a silent reservoir for *Campylobacter* spp., a critical thermotolerant foodborne pathogen [16] as well as for *Listeria* spp. [17]. The cecal microbiota of poultry represents the highest bacterial density and diversity, and its composition influences the microbiome in meat from farm to fridge [18]. The intensive use of antibiotics in poultry production has generated a critical burden of antibiotic resistance [19,20], especially in *Escherichia coli* [21] and *Campylobacter* spp. [22].

Bacteriocins have emerged as a promising alternative to antibiotics, due to their capacity to inhibit pathogenic bacteria such as *Clostridium difficile*, *Listeria*, *Escherichia coli*, and *Salmonella*, including MDR strains [23,24,25,26,27]. They exhibit a remarkable diversity in terms of biosynthetic machineries, structures, mechanisms of action, and spectra of activity. These ribosomally synthesized antimicrobial peptides are currently classified into two main categories: class I, comprising post-translationally modified peptides, and class II, comprising unmodified peptides [28,29]. Class I bacteriocins cover a great diversity of chemical families including lanthipeptides, sactipeptides, circular peptides, siderophore-peptides, and lasso peptides [30,31,32,33]. Class II bacteriocins can harbor disulfide bridges and display different secondary structures, mainly α-helix, together with β-sheet or partially extended structures [34,35]. They can be further classified as pediocin-like, two-peptide, defensin-like, leaderless, and others. The antibacterial activity of bacteriocins relies on various mechanisms including membrane pore formation, the inhibition of cell wall synthesis, and protein, deoxyribonucleic acid (DNA), or ribonucleic acid (RNA) synthesis [29]. Their spectrum of activity ranges from narrow, targeting closely related species, to broad [36]. One broad-spectrum bacteriocin is nisin, a lanthipeptide produced by certain strains of *Lactotoccus lactis*, which inhibits other lactic acid bacteria [37,38,39]. This peptide is used as a food preservative and its possible application in animal production has attracted much interest [40]. It displays a protective effect against *Clostridium* challenge in vitro [41] and in vivo in broiler chickens [42]. Another example is pediocin PA-1, a class II bacteriocin with two disulfide bridges. Produced by *Pediococcus* spp., this peptide targets many Gram-positive bacteria, notably *Listeria* [43]. In contrast, microcins are narrow-spectrum bacteriocins produced by enterobacteria, which target strains phylogenetically close to the producing bacteria [44]. One of the most studied is microcin J25 (MccJ25), a lasso peptide produced by certain strains of *Escherichia coli*, which targets *Salmonella* and *Escherichia*, through RNA polymerase inhibition [45]. MccJ25 has been shown to protect against *Salmonella* challenge in vitro [46] and in vivo in swine [47] and poultry [48].

Although different bacteriocins have been tested for their effects in vivo [49] or in vitro in models developed to mimic human or swine microbiota [35,36,37], no comparative study has been conducted to investigate their effect on broiler chicken cecal microbiota. The aim of this study was to compare the effects of bacteriocins targeting bacterial pathogens found in poultry production on the composition and metabolic activity of avian cecal microbiota. An in vitro batch fermentation model of the chicken cecal microbiota was used for this purpose. Four bacteriocins were selected based on their diversity of structure, spectrum of activity, and mechanism of action: (i) two narrow-spectrum bacteriocins targeting enterobacteria: the lasso peptide MccJ25 together with microcin E492 (MccE492), a siderophore peptide produced by *Klebsiella pneumoniae* [50,51]; and (ii) two large-spectrum bacteriocins targeting mainly Gram-positive bacteria, nisin Z and pediocin PA-1 (M31L). The effect mediated by bacteriocins was compared to that of conventional antibiotics, i.e., bacitracin, colistin, ciprofloxacin, rifampicin, and tetracycline.

## 2. Results

**Figure 1:**
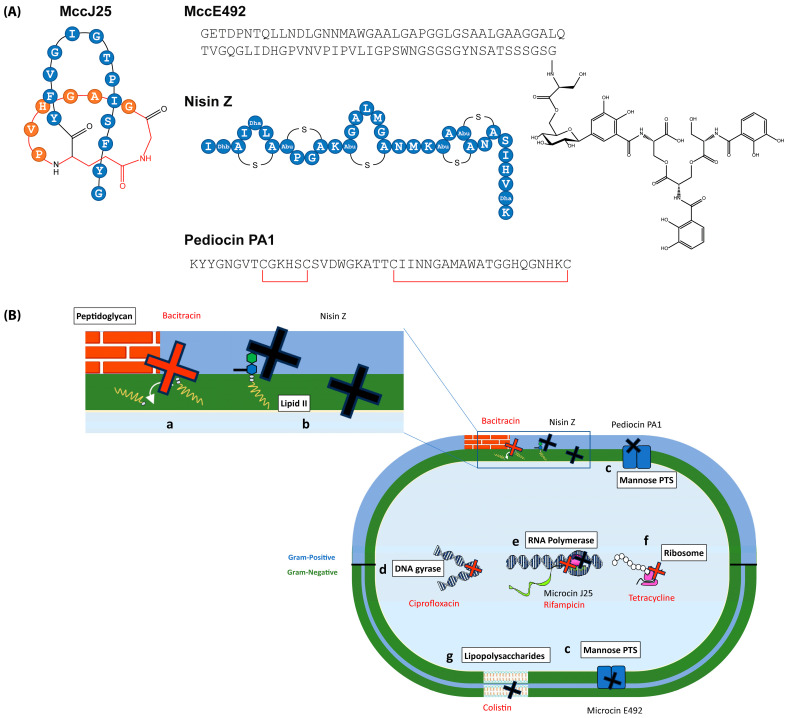
(**A**) Bacteriocins used in this study. MccJ25 is a 21 aa lasso peptide containing an *N*-terminal macrolactame ring (in orange) inside which the *C*-terminal tail of the peptide (in blue) is threaded, sterically blocked by residues Phe19 and Tyr20 located on each side of the ring. MccE92 is a 84 aa siderophore peptide where the C-terminal Ser residue is connected to three N-(2,3-dihydroxybenzoyl) units through a β-d-glucose moiety. Nisin Z is a 24 aa lanthipeptide containing (methyl)lanthionine rings resulting from thioether bridges established between dehydrated Ser/Thr residue and Cys residues. Pediocin PA-1 is a 44 aa linear peptide containing two disulfide bridges (in red). (**B**) Main targets of the antibiotics and bacteriocins used in this study. Bacitracin is a non-ribosomal cyclic polypeptide that inhibits peptidoglycan synthesis (a) by inhibiting dephosphorylation and hence the recycling of undecaprenyl pyrophosphate. It is effective (bactericidal) mainly against Gram-positive bacteria [52]. Nisin Z interacts with lipid II (b), blocking peptidoglycan biosynthesis, and forms pores in Gram-positive bacteria [53,54]. Pediocin PA-1 binds to mannose phosphotransferase (c), inducing pore formation in Gram-positive bacteria [55]. Ciprofloxacin (a bactericidal fluoroquinolone) disrupts DNA replication by targeting DNA gyrase (d) and topoisomerase IV in most Gram-negative bacteria and some Gram-positive bacteria [56]. Rifampicin is a rifamycin derivative that inhibits RNA polymerase (e) in Gram-positive and Gram-negative bacteria [57]. Tetracycline has a bacteriostatic effect against a wide range of Gram-positive and Gram-negative bacteria by binding to the 30S ribosomal subunit (f) to block protein synthesis [58]. Colistin, a polymyxin antibiotic, binds to lipopolysaccharides (g) in Gram-negative outer membranes, increasing permeability [59]. Microcin J25, its uptake mediated by FhuA, TonB, ExbD, ExbB, and SbmA, inhibits RNA polymerase [60]. MccE492, translocated though a Trojan horse strategy, is recognized by the outer membrane catecholate receptors FepA, Fiu, and/or Cir and interacts with ManYZ, the inner membrane component of the mannose permease (c), causing membrane depolarization and permeabilization [51,61].

Four bacteriocins, MccJ25, MccE49, nisin Z and pediocin PA-1 (M31L) were selected, based on diversity of structure, spectrum of activity and mechanism of action (Figure 1).

### 2.1. Production, Purification, and Antimicrobial Activity of the Different Bacteriocins

All bacteriocins were produced and purified successfully, as assessed by liquid chromatography–high-resolution mass spectrometry. All showed purity exceeding 95% and were inhibitory to the respective bacterial strain (Table 1) in agar diffusion tests (Figure S1).

### 2.2. Stability of Antimicrobial Activity over Time in Chicken Cecum Bacterial Culture

The stability of the different compounds in a batch fermentation model was evaluated by agar diffusion tests. As shown in Table 1 and Figure S2, ciprofloxacin, colistin, and tetracycline remained highly active throughout the 24 h of fermentation. Based on inhibition diameters, bacitracin was the least stable (or bioavailable) antibiotic. Among the bacteriocins, only MccJ25 showed significant inhibitory activity at 24 h, followed by pediocin PA-1 (M31L), while MccE492 revealed no activity in the complex fermentation medium at every collection time. Nisin Z only revealed a weak activity at t0.

### 2.3. Impact of the Antimicrobial Agents on Chicken Cecal Microbiota Composition In Vitro

**Figure 2:**
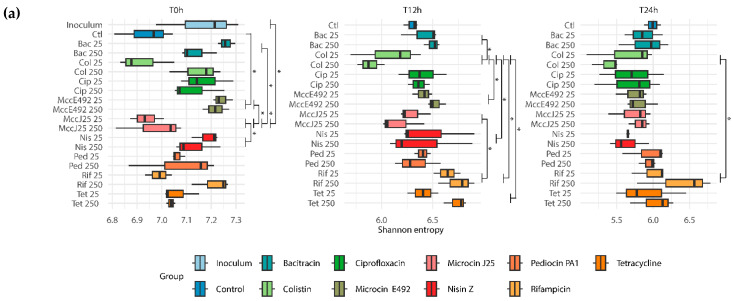

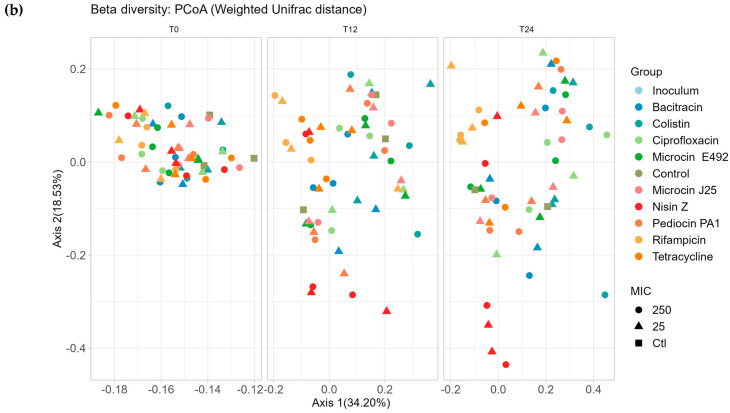
Effect of the bacteriocins and antibiotics on the diversity and richness of chicken cecum microbiota. (**a**) Alpha diversity measured as Shannon entropy in the presence of bacitracin (Bac), colistin (Col), ciprofloxacin (Cip), microcin E492 (MccE492), microcin J25 (MccJ25), nisin Z (Nis), pediocin PA-1 (M31L) (Ped), tetracycline (Tet), and rifampicin (Rif). Three time points (0 h, 12 h, and 24 h) at two concentrations (25 or 250 times the MIC) were analyzed. Tukey’s post hoc test following two-way ANOVA (*): *p* < 0.05. (**b**) Principal coordinate analysis (PCoA) of the bacterial community, with beta diversity shown in terms of Weighted Unifrac distances; PERMANOVA *p* > 0.001.

The changes in the microbial community composition over time were examined by sequencing the 16S rRNA gene and measuring alpha diversity as Shannon entropy (Figure 2a). The alpha diversity decreased with time, with an average Shannon entropy of 7.1 at t0, 6.4 at t = 12 h, and 5.8 at t = 24 h (*p* < 2 × 10^−16^). At t0, treatments with MccE492 and nisin revealed the highest diversity as compared to control. After 12 h, the treatment with colistin revealed a lower alpha diversity as compared to bacitracin, rifampicin, tetracycline, and the bacteriocins MccE492 and nisin. After 24 h, treatment with colistin induced the lowest alpha diversity, significantly different to treatment with rifampicin. The principal coordinate analysis (PCoA) of the weighted Unifrac dissimilarity matrix obtained using all samples (beta diversity; Figure 2b) revealed that nisin Z and rifampicin treatments tended to cluster separately at 24 h of fermentation.

**Figure 3:**
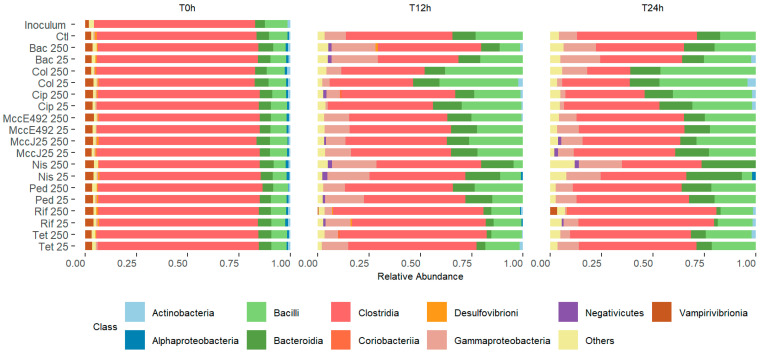
Bacterial diversity of chicken cecum microbiota in culture, determined by Illumina MiSeq-based high-throughput sequencing, in response to the presence of bacitracin (Bac), colistin (Col), ciprofloxacin (Cip), microcin E492 (MccE492), microcin J25 (MccJ25), nisin Z (Nis), pediocin PA-1 (M31L)(Ped), tetracycline (Tet), and rifampicin (Rif). The relative abundance is represented at the class level and the data are grouped into the three time points (0 h, 12 h, and 24 h).

The predominant bacterial classes identified were Clostridia, Bacilli, Bacteroidia, Gammaproteobacteria, and Actinobacteria (Figure 3). Bacitracin, colistin, ciprofloxacin, nisin Z, and pediocin PA-1 (M31L) all had dose-dependent effects at 12 h of cecal fermentation. However, this did not appear at 24 h. The relative abundance of bacilli decreased significantly in the presence of nisin at either concentration (to 0.02 and 0.05) at this point. Bacteroidia dropped markedly in the presence of rifampicin (to 0.02) or tetracycline (to 0.07 and 0.09) at 24 h. Clostridia exhibited a dose-dependent decrease in abundance in the presence of colistin or nisin. The relative abundance of Gammaproteobacteria decreased notably (to 0.03) in response to colistin at 25× MIC and to ciprofloxacin (0.03) at both concentrations. It is noteworthy that neither MccJ25 nor MccE492 induced a statistically significant alteration in the relative abundance of the identified taxa.

**Figure 4:**
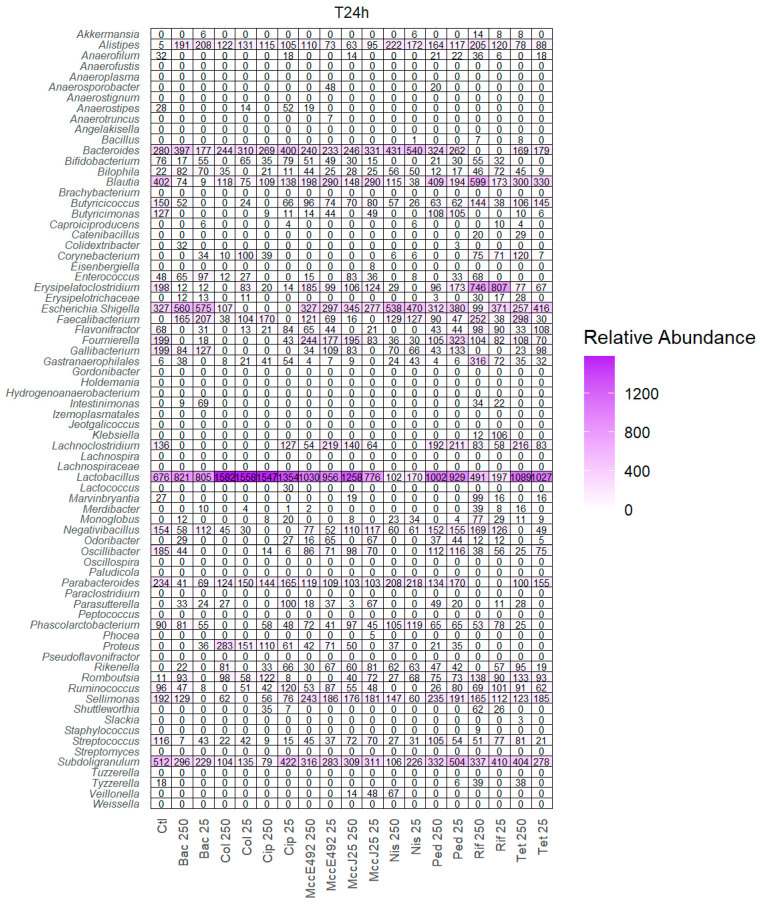
Diversity of bacterial genera in chicken cecal microbiota at 24 h of culture. The relative abundance is represented on a numerical scale and a color intensity scale. Values are the average of three biological replicates for each fermentation. All ASV sequences shared in all treatments are represented. Bacitracin (Bac), colistin (Col), ciprofloxacin (Cip), microcin E492 (MccE492), microcin J25 (MccJ25), nisin Z (Nis), pediocin PA-1 (M31L) (Ped), tetracycline (Tet), and rifampicin (Rif).

All antibiotics tested impacted certain abundant genera, in accordance with their spectrum of activity (Figure 4 and Figure S3). Bacitracin mainly impacted *Streptococcus* and *Ruminococcus* together with different Clostridia (*Lachnoclostridium*, *Erysipelatoclostridium*, and *Oscillibacter*), while promoting the genera *Escherichia*/*Shigella*. Colistin strongly impaired *Escherichia*/*Shigella* together with other Gram-negative bacteria (*Gallibacterium* and *Butyricimonas*). Ciprofloxacin impacted Gram-positive (Clostridia, *Enterococcus* and *Streptococcus*) as well as Gram-negative bacteria (*Escherichia*/*Shigella* and *Gallibacterium*). Rifampicin mainly impacted the genera *Bacteroides* and *Parabacteroides*. Tetracycline mainly affected the Gram-negative bacteria *Gallibacterium* and the Gram-positive bacteria *Phascolarctobacterium*.

The relative abundance of *Escherichia* dropped in the presence of colistin or ciprofloxacin, but was not affected by the tested bacteriocins. After 24 h, the genera *Enterococcus* was reduced in the presence of nisin Z and MccE492 and the highest concentration of pediocin PA-1 (M31L), as well as in the presence of the antibiotics ciprofloxacin and tetracycline and the lowest concentration of rifampicin (Figure 4). Among the four bacteriocins, nisin Z had the most impact on microbiota composition, affecting not only *Enterococcus* but also various genera such as *Lachnoclostridium*, *Erysipelatoclostridium*, *Ruminococcus*, *Oscillibacter, Lactobacillus, Bifidobacterium*, and *Butyricimonas*. Pediocin PA-1 (M31L) had a moderate effect, altering mainly *Enterococcus* together with *Ruminococcus*, *Bifidobacterium*, and *Gallibacterium* only at the highest concentration tested. MccJ25 and MccE492 revealed only minor effects on the microbiota composition: MccE492 mainly affected *Enterococcus* while MccJ25 mainly affected *Gallibacterium*.

### 2.4. Impact of Antimicrobial Agents on Short-Chain Fatty Acid Metabolism by Cecal Microbiota

**Figure 5:**
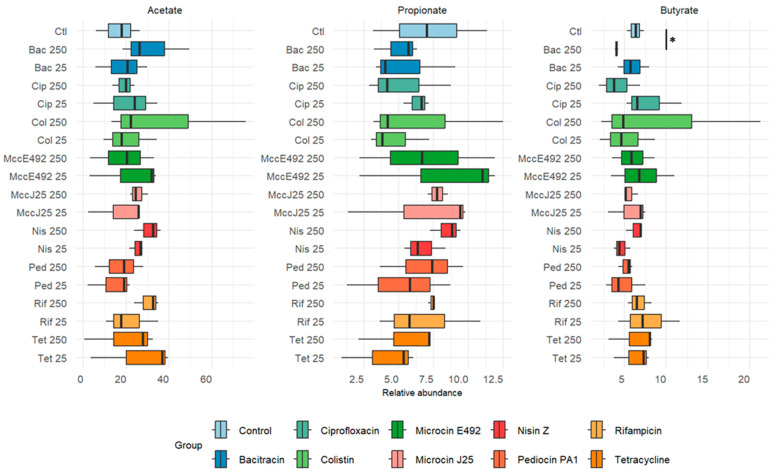
Relative abundance of short-chain fatty acids in chicken cecum microbiota cultured in penicillin bottles for 24 h. Control (Ctl), Bacitracin (Bac), colistin (Col), ciprofloxacin (Cip), microcin E492 (Mcc E492), microcin J25 (MccJ25), nisin Z (Nis), and pediocin PA-1 (M31L) (Ped). Kruskal–Wallis pairwise test (*): *p* < 0.05.

The short-chain fatty acid content of the fermentation broth supernatant is shown in Figure 5. Butyrate levels showed a significant decrease in response to bacitracin. No other significant difference was observed, due to high variability between replicates. Acetate production reached maximal ranges in response to bacitracin, nisin Z, and rifampicin at 250 times the MIC, while butyrate levels tended to decrease in response to colistin at the highest concentration and to nisin and pediocin PA-1 (M31L) at the lowest concentration.

## 3. Discussion

Evaluating the potential of bacteriocins as an alternative to antibiotics in livestock production requires a deep and comprehensive study of the behavior, stability, and activity of these molecules under gastrointestinal conditions as well as their impact on the microbiota composition and activity in comparison with antibiotics. In this study, each antimicrobial compound was tested at 25 times the MIC and at 250 times the MIC, two concentrations based on our previous studies and within the range of concentrations that can be used for various applications in animals.

The batch fermentation model used in this study allows researchers to reproduce in vitro the cecal microbiota. Its composition is in agreement with that reported from cecal samples collected on boiler chickens from intensive production systems [65]. Batch fermentation constitutes an interesting model to screen multiple conditions in vitro [66]. However, the host component is absent in this model and the microbiota stability over time is limited due to nutrient depletion and the absence of pH control. This is illustrated by the decrease in alpha diversity over time observed in this study. Such a simple model is appropriate to screen multiple treatments on a limited time scale (24 h).

The residual activity of bacteriocins measured by an agar diffusion assay over a 24 h period in the complex cecal fermentation medium was used as an indicator of their stability and bioavailability. The interpretation of the activities measured should be interpreted with caution. Given the low sensitivity of the test, the absence of an inhibition zone does not necessarily mean the complete degradation of the compound. For example, nisin showed no inhibitory activity against its test organism but it did show a significant effect on cecal microbiota composition. This suggests that nisin remains partially active and therefore influences the cecal microbiota. On the other hand, microcin J25, which showed very high stability as the inhibitor of a single test organism, had no effect on the microbiota composition. The instability of a given compound can be compensated for by using concentrations well above the MIC and therefore obtaining significant residual activity. More sensitive approaches, such as a quantitative microtitration assay or analytical methods such as Liquid Chromatography-Mass Spectrometry (LC-MS), are still needed for the more accurate and sensitive detection and quantification of these bacteriocins in such complex media.

Our results show that MccJ25 remains active under in vitro cecal conditions over the 24 h of batch fermentation. The lasso structure of MccJ25 confers the peptide with remarkable resistance to proteases, high temperatures, and extreme pH [33,45]. MccJ25 has previously been shown to be stable in gastric conditions and partially hydrolyzed in the loop region in duodenal conditions, generating degradation products consisting of two-peptides non-covalently associated through the steric hindrance provided by the threaded structure [67,68]. Such hydrolysis products have also been detected in a continuous culture model mimicking swine microbiota [69]. This ensemble of observations confirms the interest of MccJ25 as promising alternative to antibiotics to protect poultry from pathogenic enterobacteria. It is also consistent with several in vivo studies showing that MccJ25 displayed prolonged antimicrobial activity in a murine infection model [70] and protected against *Salmonella* challenge in swine [47] and poultry [48]. The absence of modulation of the cecal microbiota composition by MccJ25 could be explained by its narrow spectrum of activity, which is mainly driven by the mechanism of uptake in the target bacteria [71,72]. The difference in the sequence of the outer membrane receptor FhuA can result in contrasted sensitivities in *Escherichia coli* and *Salmonella enterica*. Thus, MccJ25 would modulate the composition of *Escherichia coli* and *Salmonella enterica* at the strain level, without impacting the overall density of the considered genera.

MccE492 has been studied primarily for its potential to induce apoptosis in human cell lines and for possible antitumor effects against colorectal cancer [61,73]. To the best of our knowledge, the present study is the first to evaluate the activity of MccE492 under chicken cecal conditions. We demonstrated that this bacteriocin is not active against the tested indicative strain *Salmonella enterica* when introduced in the cecal fermentation model. Other siderophore microcins (namely, microcins H47 and M) produced by the probiotic strain *E. coli* Nissle have been shown to limit the expansion of competing *Enterobacteriaceae* including pathogens and pathobionts in mice with intestinal inflammation [74]. The authors of this study suggest that for the in vivo application of such microcins, using the producing strain as a probiotic rather than the purified peptide might be a more effective strategy, thereby helping to counter gastrointestinal degradation. This alternative can be proposed also for microcin E492, given its weak stability/bioavailability in the cecal microbiota conditions. The unexpected vulnerability of *Enterococcus* to MccE492 should be confirmed by antibacterial assays in aerobic versus anaerobic conditions, as differences depending on energy metabolism have already been reported in the sensibility of *Enterococcus faecium* to polymyxin B [75].

In chicken cecal conditions, pediocin PA-1 (M31L) conserved a weak activity over the 24 h incubation and displayed only a moderate impact on the microbiota composition at the highest concentration tested. In contrast, this bacteriocin was found to be sensitive to gastrointestinal conditions, losing its antibacterial activity completely in conditions mimicking the small intestine [67,76]. Previous studies performed in vitro and in vivo showed that pediocin PA-1 produced by *Pediococcus acidilactici* or the introduction of a probiotic producing strain [77] did not perturb much the gut microbiota [37,78,79], in line with our observations with the M31L variant.

The most drastic impact on the microbiota composition was observed for nisin Z, although its activity against *Listeria* was reduced in the cecal conditions. Recently, nisin formulated as powder was shown to tolerate transit through the porcine gastrointestinal tract intact when administered at 150 mg/kg body weight [41] whereas nisin Z at lower concentrations was degraded completely in a gastrointestinal tract simulator [67]. In vivo poultry studies have shown that nisin lowers the number of bacterial cells of the genera *Lactobacillus, Bacteroides, Enterococcus*, and *Clostridium* [80,81]. Our present study is in agreement with these observations, except for the genera *Bacteroides*, which in our in vitro conditions tended to be promoted in the presence of nisin Z. The decrease in *Lactobacillus* is consistent with the spectrum of action of nisin, which inhibits the growth of bacteria closely related to the producing strain [82]. Consistent with a broiler chicken study in vivo [81], we found that nisin Z tented to increase acetate levels.

Regarding antibiotics, the observed effects on the major genera corresponded generally to expected spectra of activity. The impact of bacitracin on poultry gut microbiota has been studied in several in vivo trials [83,84]. Significant influence on fecal microbiota composition were observed, but no marked effect on microbial richness. Our results corroborate this observation. Tetracyclines have been used in poultry production for several decades, and their effects on the fecal microbiome have been studied in vivo [85]. At therapeutic doses in broiler chickens, chlortetracycline was found to reduce Proteobacteria, in particular *Escherichia* and *Shigella* genera. In our chicken cecal batch fermentation, we also observed a decrease in certain Gammaproteobacteria, such as *Gallibacterium* class, but no effect on *Escherichia*/*Shigela*. This lack of activity in the cecal microbiota used in our study could result from acquired resistance, as high resistance to tetracycline has been reported in *Escherichia coli* isolates from broiler chicken [86,87]. There is limited specific information about the effect of rifampicin and ciprofloxacin on poultry cecal microbiota. These antibiotics have not been used widely in poultry production, which may explain the lack of studies of their effects.

Overall, this study highlighted that bacteriocins with diverse structures, mechanisms, and spectra of activity result in contrasted effects on the chicken cecal microbiota. Nisin Z, which show a dual mechanism of action and the widest spectrum of activity, displays the most important alteration of the cecal microbiota composition, but appears as a promising strategy to combat colonization by pathogenic Enterococci or Clostridia. In contrast, MccJ25, which displays a narrow-spectrum activity against Enterobacteria, does not significantly modify the cecal microbiota composition and appears as a promising strategy to combat colonization by *Salmonella*. Such bacteriocins could permit researchers to reduce the use of antibiotics in animal production and thus the spread of antibiotic resistance, while protecting from important pathogens through a more targeted approach. An important aspect to examine further is their propensity to induce resistance. The dual mechanism of action of nisin has limited the emergence of resistance despite its wide use as a food preservative since the 1950s [88]. Another important aspect to explore further is the long-term effects of bacteriocins on gut health in live animals. This work permits researchers to select for future work the bacteriocins that would be most valuable in light of their stability and bioavailability, i.e., microcin J25 and pediocin PA-1 (M31L).

## 4. Materials and Methods

### 4.1. Bacteriocin Production

MccE492 was produced heterologously in *E. coli* MC4100 harboring the plasmid pJAM229 [50] grown in M63 minimal medium. It was loaded directly from the culture supernatant onto a 35 cc OASIS cartridge (Waters, Milford, MA, USA) and eluted into a mobile phase of H_2_O + 0.1% formic acid with an acetonitrile gradient from 20% to 80%. The modified form of MccE492, with its C-terminus connected to a glucose linked to three N-(2,3-dihydroxybenzoyl) units, was purified from the 40% acetonitrile fraction by Reverse Phase High Performance Liquid Chromatography (RP-HPLC), as described previously [63].

MccJ25 was produced heterologously in *E. coli* MC4100 harboring the plasmid pTUC 202 [89] grown in M63 minimal medium. It was loaded directly from the culture supernatant onto a 35 cc C8 cartridge and eluted into the 30% acetonitrile fraction and purified by RP-HPLC, as described previously [64].

Pediocin PA-1 (M31L) was prepared by standard solid-phase peptide synthesis and purified by RP-HPLC, as described previously [55].

Nisin Z was isolated from a commercial solution (Niseen^®^-S) purchased from Fromagex (Rimouski, QC, Canada) and purified by successive centrifugations [90].

The purified peptides were analyzed by high-resolution LC-MS on an Ultimate 3000-RSLC system (Thermo Scientific, Waltham, MA, USA) connected to an electrospray ionization quadrupole time-of-flight instrument (Maxis II ETD, Bruker Daltonics, Billerica, MA, USA). Resolution was achieved using an RSLC Polar Advantage II Acclaim column (2.2 μm, 120 Å, 2.1 × 100 mm, Thermo Scientific, Waltham, MA, USA) using a linear gradient (2–80% in 20 min) of mobile phases FA 0.1% and LC-MS grade acetonitrile (ACN) + FA 0.08%, at 300 μL min^−1^.

### 4.2. Antibiotics

The broad-spectrum antibiotics bacitracin, ciprofloxacin, colistin, rifampicin, and tetracycline were purchased from Sigma-Aldrich (Saint-Louis, MI, USA).

### 4.3. Cecal Bacteria Collection

For each biological replicate, the gastrointestinal (GI) tracts of four freshly slaughtered 35-day-old broiler chickens were obtained from the Exceldor Group abattoir (Exceldor Group, Saint-Anselme, QC, Canada). Upon collection, samples were placed in a sterile container with an Oxoid AnaeroGen strip (Thermo Scientific, Waltham, MA, USA) and transported to the laboratory within 1 h. The GI tracts were placed in an anaerobic chamber (Coy Laboratories, Ann Arbor, MI, USA). Feces from the ileum and cecal contents were combined to inoculate the culture medium.

### 4.4. Batch Fermentation

Sterile Viande Levure (VL) medium (16 mL in 50 mL penicillin bottles), supplemented with Tween 80, bile salts, minerals, vitamins, porcine mucin (2.0 g/L), fructo-oligosaccharide (2.5 g/L), and citrus pectin (2.5 g/L), as described previously [91], and adjusted to pH 6 with 5 M NaOH, was inoculated with 8 mL fecal slurry (10% inoculum, *w*/*v*). The antimicrobial compound was added in a volume of 1 mL to obtain 25 or 250 times the minimal inhibitory concentration (Table 1).

To ensure anaerobic conditions, the bottles were inoculated and sealed in an oxygen-free chamber. They were incubated at 41 °C and 80 rpm on an orbital shaker for up to 24 h. For each experimental treatment, samples were collected at 0, 12, and 24 h and kept at −80 °C until analysis.

### 4.5. Agar Well Diffusion Tests

The inhibitory activity of each antimicrobial agent was assessed qualitatively in terms of inhibition zones around agar wells. Briefly, 80 μL of sample was added to wells in pour plates (25 mL) seeded with 250 µL of overnight culture of indicator strain. Following 18 h of incubation at the appropriate temperature, inhibition diameters were measured. Photographs were taken using ChemiDoc XRS (Bio-Rad, Hercules, CA, USA).

### 4.6. DNA Extraction and 16S Gene Sequencing

Fermentation broth was centrifuged for 8 min at 12,000 x g and the pellets were stored at −80 °C. DNA was extracted therefrom using the QIAamp PowerFecal Pro DNAKit (Qiagen, Hilden, Germany). DNA quality and concentration were assessed using a NanoDrop 1000 spectrophotometer (Thermo Scientific, Wilmington, DE, USA).

The primer pairs 341F (5′-TCGTCGGCAGCGTCAGATGTGTATAAGAGACAGCTA CGGGNGGCWGCAG-3′) and 805R (5′-GTCTCGTGGGCTCGGAGATGTGTATAAGAG ACAGGACTACHVGGGTATCTAATCC-3′) were employed to amplify the V3-V4 hypervariable region of the 16 S rRNA gene. The amplicons were sequenced at the Centre de recherche du CHU de Québec-Université Laval using Illumina MiSeq paired-end technology. Sequences were analyzed in Ubuntu terminal using the Qiime2 version 2022.2 [92]. DADA2 plugin was employed to infer the amplicon sequence variants (ASVs). Sequence reads underwent filtering and trimming (trim-left-f 5, trim-left-r 5, trunc-len-f 280, and trunc-len-r 240). The filtered reads were then denoised, and chimeric sequences were removed [93]. Taxonomic affiliations were obtained by the sklearn-based classifier (SILVA release 138 database trained for the V3-V4 region) employing default parameters for taxonomic assignment [94]. Principal coordinate analysis (PCoA) plots based on weighted UniFrac distances and the Shannon diversity index were generated using ggplot2 version 3.5.1 [95] in R version 4.4.0 [96]. To compare the effects of treatment and time on alpha diversity, we used two-way ANOVA followed by Tukey’s multiple comparisons test. Bacterial compositions were compared using PERMANOVA.

### 4.7. Quantification of Short-Chain Fatty Acids

Fermentation supernatants were extracted with an equal volume of acetonitrile. Acetate, propionate, and butyrate were quantified using a ThermoFisher (Waltham, MA, USA) Ultimate 3000 UHPLC system equipped with a UV detector at 210 nm. Compounds were separated on a 250 × 4.6 mm Rezex ROA-Organic acid LC column (Phenomenex Torrance, CA, USA) at a flow rate of 0.4 mL min^−1^ and a column temperature of 85 °C. Separation was isocratic with a mobile phase of HPLC-grade water acidified with 5 mM H_2_SO_4_. The chromatograms were analyzed with ThermoFisher Chromeleon 7(Waltham, MA, USA), and organic acid concentrations in the fermentation supernatants were determined by measuring peak areas. Kruskal-Wallis test, followed by post-hoc pairwise comparisons using Dunn’s test with Bonferroni correction, were used to compare the effect of treatments for each organic acid.

## 5. Conclusions

This study is the first to compare, under the same experimental conditions, the effect of four bacteriocins and of diverse antibiotics active against Gram-positive and/or Gram-negative poultry pathogens on the composition and metabolic activity of chicken cecal microbiota. The use of advanced metagenomic and metabolomic approaches allowed us to demonstrate that the impact of bacteriocins strongly relies on their spectrum of activity and differs strongly between bacteriocins produced by Gram-negative and Gram-positive bacteria. These results provide support for examining bacteriocins as alternatives to antibiotics and possibly their use as a new generation of antimicrobial agents in livestock production.

## Figures and Tables

**Table 1:** Minimum inhibitory concentration (MIC) of the antimicrobial compounds tested (provided in µg/mL and in µmol/L) and residual activities over time in the cecal fermentation medium, after the introduction of the compounds at 25 (top) or 250 times (bottom) the minimal inhibitory concentration (MIC). The indicative strains were *Streptococcus pyogenes* RBL4, for bacitracin, *Listeria ivanovii* HPB28 for nisin Z and pediocin PA-1 (M31L), and *Salmonella enterica* subsp. *enterica* serotype Newport ATCC 6962 for all others. The inhibitory activity is represented by the size of inhibition zone: (+++), (++), (+), and (−) when absent (see agar plates in Figure S2).

Compound	MIC (µg/mL)	MIC (µM)	Sensitive Strain	Residual Activity				Reference
				0 h	6 h	12 h	24 h	
Bacitracin	1.50	0.17	*C. perfringens*	+ +	+ +	+ +	+ +	[62]
Ciprofloxacin	0.19	0.60	*S. enterica*	+++ +++	+++ +++	+++ +++	+++ +++	[63]
Colistin	0.80	0.69	*S. enterica*	++ ++	++ ++	++ ++	++ ++	[63]
Microcin E492	1.56	0.18	*S. enterica*	− −	− −	− −	− −	[63]
Microcin J25	0.06	0.03	*S. enterica*	+ ++	+ ++	+ ++	+ ++	[63]
Nisin Z	1.56	0.18	*L. ivanovii*	+ −	− −	− −	− −	[64]
Pediocin PA-1 (M31L)	0.09	0.12	*L. ivanovii*	+ +	+ +	+ +	+ +	[64]
Rifampicin	0.39	0.47	*S. enterica*	− −	− −	− −	− −	[63]
Tetracycline	4.00	9.00	*S. enterica*	+++ +++	+++ +++	+++ +++	+++ +++	[63]

## Data Availability

Data is contained within the article and Supplementary Materials.

## Supplementary Materials

The following supporting information can be downloaded at https://www.mdpi.com/article/10.3390/ijms26020755/s1.

## References

[1] Oliveira N.A., Gonçalves B.L., Lee S.H., Caf O., Corassin C.H. (2020). Use of Antibiotics in Animal Production and Its Impact on Human Health. J. Food Chem. Nanotechnol..

[2] Hosain M.Z., Kabir S.M.L., Kamal M.M. (2021). Antimicrobial Uses for Livestock Production in Developing Countries. Vet. World.

[3] Shallcross L.J., Davies D.S.C. (2014). Antibiotic Overuse: A Key Driver of Antimicrobial Resistance. Br. J. Gen. Pract..

[4] White A., Hughes J.M. (2019). Critical Importance of a One Health Approach to Antimicrobial Resistance. EcoHealth.

[5] Aidara-Kane A., Angulo F.J., Conly J.M., Minato Y., Silbergeld E.K., McEwen S.A., Collignon P.J., Balkhy H., Collignon P., Conly J. (2018). World Health Organization (WHO) Guidelines on Use of Medically Important Antimicrobials in Food-Producing Animals. Antimicrob. Resist. Infect. Control.

[6] Van T.T.H., Yidana Z., Smooker P.M., Coloe P.J. (2020). Antibiotic Use in Food Animals Worldwide, with a Focus on Africa: Pluses and Minuses. J. Glob. Antimicrob. Resist..

[7] Mulchandani R., Wang Y., Gilbert M., Boeckel T.P.V. (2023). Global Trends in Antimicrobial Use in Food-Producing Animals: 2020 to 2030. PLoS Glob. Public Health.

[8] Allen H.K., Levine U.Y., Looft T., Bandrick M., Casey T.A. (2013). Treatment, Promotion, Commotion: Antibiotic Alternatives in Food-Producing Animals. Trends Microbiol..

[9] Stoica C., Cox G. (2021). Old Problems and New Solutions: Antibiotic Alternatives in Food Animal Production. Can. J. Microbiol..

[10] Rahman M.R.T., Fliss I., Biron E. (2022). Insights in the Development and Uses of Alternatives to Antibiotic Growth Promoters in Poultry and Swine Production. Antibiotics.

[11] Chowdhury M.A.H., Ashrafudoulla M., Mevo S.I.U., Mizan M.F.R., Park S.H., Ha S.-D. (2023). Current and Future Interventions for Improving Poultry Health and Poultry Food Safety and Security: A Comprehensive Review. Compr. Rev. Food Sci. Food Saf..

[12] Kabir S.M.L. (2010). Avian Colibacillosis and Salmonellosis: A Closer Look at Epidemiology, Pathogenesis, Diagnosis, Control and Public Health Concerns. Int. J. Environ. Res. Public Health.

[13] Mora Z.V.-d.l., Macías-Rodríguez M.E., Arratia-Quijada J., Gonzalez-Torres Y.S., Nuño K., Villarruel-López A. (2020). Clostridium Perfringens as Foodborne Pathogen in Broiler Production: Pathophysiology and Potential Strategies for Controlling Necrotic Enteritis. Animals.

[14] Souillard R., Laurentie J., Kempf I., Le Caër V., Le Bouquin S., Serror P., Allain V. (2022). Increasing Incidence of *Enterococcus*-Associated Diseases in Poultry in France over the Past 15 Years. Vet. Microbiol..

[15] Akil L., Ahmad H.A. (2019). Quantitative Risk Assessment Model of Human Salmonellosis Resulting from Consumption of Broiler Chicken. Diseases.

[16] Sahin O., Kassem I.I., Shen Z., Lin J., Rajashekara G., Zhang Q. (2015). Campylobacter in Poultry: Ecology and Potential Interventions. Avian Dis..

[17] Rothrock M.J., Davis M.L., Locatelli A., Bodie A., McIntosh T.G., Donaldson J.R., Ricke S.C. (2017). Listeria Occurrence in Poultry Flocks: Detection and Potential Implications. Front. Vet. Sci..

[18] Marmion M., Ferone M.T., Whyte P., Scannell A.G.M. (2021). The Changing Microbiome of Poultry Meat; from Farm to Fridge. Food Microbiol..

[19] Agyare C., Boamah V.E., Zumbi C.N., Osei F.B. (2018). Antibiotic Use in Poultry Production and Its Effects on Bacterial Resistance. Antimicrobial Resistance—A Global Threat.

[20] Islam M.A., Bose P., Rahman M.Z., Muktaruzzaman M., Sultana P., Ahamed T., Khatun M.M. (2024). A Review of Antimicrobial Usage Practice in Livestock and Poultry Production and Its Consequences on Human and Animal Health. J. Adv. Vet. Anim. Res..

[21] Alonso C.A., Zarazaga M., Ben Sallem R., Jouini A., Ben Slama K., Torres C. (2017). Antibiotic Resistance in Escherichia Coli in Husbandry Animals: The African Perspective. Lett. Appl. Microbiol..

[22] Yang Y., Feye K.M., Shi Z., Pavlidis H.O., Kogut M., Ashworth A.J., Ricke S.C. (2019). A Historical Review on Antibiotic Resistance of Foodborne Campylobacter. Front. Microbiol..

[23] Rea M.C., Sit C.S., Clayton E., O’Connor P.M., Whittal R.M., Zheng J., Vederas J.C., Ross R.P., Hill C. (2010). Thuricin CD, a Posttranslationally Modified Bacteriocin with a Narrow Spectrum of Activity Against *Clostridium difficile*. Proc. Natl. Acad. Sci. USA.

[24] Jiang H., Li P., Gu Q. (2016). Heterologous Expression and Purification of Plantaricin Nc8, a Two-Peptide Bacteriocin against *Salmonella* Spp. from *Lactobacillus Plantarum* Zj316. Protein Expr. Purif..

[25] Khorshidian N., Khanniri E., Mohammadi M., Mortazavian A.M., Yousefi M. (2021). Antibacterial Activity of Pediocin and Pediocin-Producing Bacteria against *Listeria Monocytogenes* in Meat Products. Front. Microbiol..

[26] Xiang Y.-Z., Zhang Y.-M., Liu Y.-Y., Zhang M., Lin L.-B., Zhang Q.-L. (2021). Purification, Characterization, and Antibacterial and Antibiofilm Activity of a Novel Bacteriocin against *Salmonella* Enteritidis. Food Control.

[27] Xin W.-G., Wu G., Ying J.-P., Xiang Y.-Z., Jiang Y.-H., Deng X.-Y., Lin L.-B., Zhang Q.-L. (2023). Antibacterial Activity and Mechanism of Action of Bacteriocin *Lfx01* against *Staphylococcus aureus* and *Escherichia coli* and Its Application on Pork Model. Meat Sci..

[28] Reuben R.C., Torres C. (2024). Bacteriocins: Potentials and Prospects in Health and Agrifood Systems. Arch. Microbiol..

[29] Sugrue I., Ross R.P., Hill C. (2024). Bacteriocin Diversity, Function, Discovery and Application as Antimicrobials. Nat. Rev. Microbiol..

[30] Repka L.M., Chekan J.R., Nair S.K., van der Donk W.A. (2017). Mechanistic Understanding of Lanthipeptide Biosynthetic Enzymes. Chem. Rev..

[31] Gabrielsen C., Brede D.A., Nes I.F., Diep D.B. (2014). Circular Bacteriocins: Biosynthesis and Mode of Action. Appl. Environ. Microbiol..

[32] Vassiliadis G., Peduzzi J., Zirah S., Thomas X., Rebuffat S., Destoumieux-Garzón D. (2007). Insight into Siderophore-Carrying Peptide Biosynthesis: Enterobactin Is a Precursor for Microcin E492 Posttranslational Modification. Antimicrob. Agents Chemother..

[33] Wilson K.-A., Kalkum M., Ottesen J., Yuzenkova J., Chait B.T., Landick R., Muir T., Severinov K., Darst S.A. (2003). Structure of Microcin J25, a Peptide Inhibitor of Bacterial RNA Polymerase, Is a Lassoed Tail. J. Am. Chem. Soc..

[34] Cui Y., Zhang C., Wang Y., Shi J., Zhang L., Ding Z., Qu X., Cui H. (2012). Class IIa Bacteriocins: Diversity and New Developments. Int. J. Mol. Sci..

[35] Antoshina D.V., Balandin S.V., Ovchinnikova T.V. (2022). Structural Features, Mechanisms of Action, and Prospects for Practical Application of Class II Bacteriocins. Biochem. Mosc..

[36] Cotter P.D., Ross R.P., Hill C. (2013). Bacteriocins—A Viable Alternative to Antibiotics?. Nat. Rev. Microbiol..

[37] Le Blay G., Hammami R., Lacroix C., Fliss I. (2012). Stability and Inhibitory Activity of Pediocin PA-1 against *Listeria* sp. in Simulated Physiological Conditions of the Human Terminal Ileum. Probiotics Antimicrob. Proteins.

[38] Field D., Fernandez de Ullivarri M., Ross R.P., Hill C. (2023). After a Century of Nisin Research—Where Are We Now?. FEMS Microbiol. Rev..

[39] Charest A.M., Reed E., Bozorgzadeh S., Hernandez L., Getsey N.V., Smith L., Galperina A., Beauregard H.E., Charest H.A., Mitchell M. (2024). Nisin Inhibition of Gram-Negative Bacteria. Microorganisms.

[40] Gharsallaoui A., Oulahal N., Joly C., Degraeve P. (2016). Nisin as a Food Preservative: Part 1: Physicochemical Properties, Antimicrobial Activity, and Main Uses. Crit. Rev. Food Sci. Nutr..

[41] O’Reilly C., Grimaud G.M., Coakley M., O’Connor P.M., Mathur H., Peterson V.L., O’Donovan C.M., Lawlor P.G., Cotter P.D., Stanton C. (2023). Modulation of the Gut Microbiome with Nisin. Sci. Rep..

[42] Yuan H., Bai G., Lin Y., Yu X., Yang Q., Dou R., Sun H., Zhao Z., Li Z., Chen Z. (2024). Effects of Dietary Nisin on Growth Performance, Immune Function, and Gut Health of Broilers Challenged by Clostridium Perfringens. J. Anim. Sci..

[43] Rodríguez J.M., Martínez M.I., Kok J. (2002). Pediocin PA-1, a Wide-Spectrum Bacteriocin from Lactic Acid Bacteria. Crit. Rev. Food Sci. Nutr..

[44] Telhig S., Ben Said L., Zirah S., Fliss I., Rebuffat S. (2020). Bacteriocins to Thwart Bacterial Resistance in Gram Negative Bacteria. Front. Microbiol..

[45] Baquero F., Beis K., Craik D.J., Li Y., Link A.J., Rebuffat S., Salomón R., Severinov K., Zirah S., Hegemann J.D. (2024). The Pearl Jubilee of Microcin J25: Thirty Years of Research on an Exceptional Lasso Peptide. Nat. Prod. Rep..

[46] Naimi S., Zirah S., Taher M.B., Theolier J., Fernandez B., Rebuffat S.F., Fliss I. (2020). Microcin J25 Exhibits Inhibitory Activity Against Salmonella Newport in Continuous Fermentation Model Mimicking Swine Colonic Conditions. Front. Microbiol..

[47] Yu H.T., Ding X.L., Li N., Zhang X.Y., Zeng X.F., Wang S., Liu H.B., Wang Y.M., Jia H.M., Qiao S.Y. (2017). Dietary Supplemented Antimicrobial Peptide Microcin J25 Improves the Growth Performance, Apparent Total Tract Digestibility, Fecal Microbiota, and Intestinal Barrier Function of Weaned Pigs. J. Anim. Sci..

[48] Wang G., Song Q., Huang S., Wang Y., Cai S., Yu H., Ding X., Zeng X., Zhang J. (2020). Effect of Antimicrobial Peptide Microcin J25 on Growth Performance, Immune Regulation, and Intestinal Microbiota in Broiler Chickens Challenged with *Escherichia Coli* and *Salmonella*. Animals.

[49] Benítez-Chao D.F., León-Buitimea A., Lerma-Escalera J.A., Morones-Ramírez J.R. (2021). Bacteriocins: An Overview of Antimicrobial, Toxicity, and Biosafety Assessment by in Vivo Models. Front. Microbiol..

[50] Thomas X., Destoumieux-Garzón D., Peduzzi J., Afonso C., Blond A., Birlirakis N., Goulard C., Dubost L., Thai R., Tabet J.-C. (2004). Siderophore Peptide, a New Type of Post-Translationally Modified Antibacterial Peptide with Potent Activity. J. Biol. Chem..

[51] Huang K., Zeng J., Liu X., Jiang T., Wang J. (2021). Structure of the Mannose Phosphotransferase System (Man-PTS) Complexed with Microcin E492, a Pore-Forming Bacteriocin. Cell Discov..

[52] Smith J.L., Weinberg E.D. (1962). Mechanisms of Antibacterial Action of Bacitracin. Microbiology.

[53] Prince A., Sandhu P., Ror P., Dash E., Sharma S., Arakha M., Jha S., Akhter Y., Saleem M. (2016). Lipid-II Independent Antimicrobial Mechanism of Nisin Depends on Its Crowding and Degree of Oligomerization. Sci. Rep..

[54] Santos J.C.P., Sousa R.C.S., Otoni C.G., Moraes A.R.F., Souza V.G.L., Medeiros E.A.A., Espitia P.J.P., Pires A.C.S., Coimbra J.S.R., Soares N.F.F. (2018). Nisin and Other Antimicrobial Peptides: Production, Mechanisms of Action, and Application in Active Food Packaging. Innov. Food Sci. Emerg. Technol..

[55] Bédard F., Hammami R., Zirah S., Rebuffat S., Fliss I., Biron E. (2018). Synthesis, Antimicrobial Activity and Conformational Analysis of the Class IIa Bacteriocin Pediocin PA-1 and Analogs Thereof. Sci. Rep..

[56] Shariati A., Arshadi M., Khosrojerdi M.A., Abedinzadeh M., Ganjalishahi M., Maleki A., Heidary M., Khoshnood S. (2022). The Resistance Mechanisms of Bacteria against Ciprofloxacin and New Approaches for Enhancing the Efficacy of This Antibiotic. Front. Public Health.

[57] Campbell E.A., Korzheva N., Mustaev A., Murakami K., Nair S., Goldfarb A., Darst S.A. (2001). Structural Mechanism for Rifampicin Inhibition of Bacterial RNA Polymerase. Cell.

[58] Chopra I., Roberts M. (2001). Tetracycline Antibiotics: Mode of Action, Applications, Molecular Biology, and Epidemiology of Bacterial Resistance. Microbiol. Mol. Biol. Rev..

[59] Andrade F.F., Silva D., Rodrigues A., Pina-Vaz C. (2020). Colistin Update on Its Mechanism of Action and Resistance, Present and Future Challenges. Microorganisms.

[60] Ducasse R., Yan K.-P., Goulard C., Blond A., Li Y., Lescop E., Guittet E., Rebuffat S., Zirah S. (2012). Sequence Determinants Governing the Topology and Biological Activity of a Lasso Peptide, Microcin J25. ChemBioChem.

[61] Lagos R., Tello M., Mercado G., Garcia V., Monasterio O. (2009). Antibacterial and Antitumorigenic Properties of Microcin E492, a Pore- Forming Bacteriocin. Curr. Pharm. Biotechnol..

[62] Charlebois A., Jacques M., Archambault M. (2014). Biofilm Formation of *Clostridium perfringens* and Its Exposure to Low-Dose Antimicrobials. Front. Microbiol..

[63] Telhig S., Ben Said L., Torres C., Rebuffat S., Zirah S., Fliss I. (2022). Evaluating the Potential and Synergetic Effects of Microcins against Multidrug-Resistant Enterobacteriaceae. Microbiol. Spectr..

[64] Soltani S., Biron E., Said L.B., Subirade M., Fliss I. (2022). Bacteriocin-Based Synergetic Consortia: A Promising Strategy to Enhance Antimicrobial Activity and Broaden the Spectrum of Inhibition. Microbiol. Spectr..

[65] Marcolla C.S., Ju T., Lantz H.L., Willing B.P. (2023). Investigating the Cecal Microbiota of Broilers Raised in Extensive and Intensive Production Systems. Microbiol. Spectr..

[66] Pérez-Burillo S., Molino S., Navajas-Porras B., Valverde-Moya Á.J., Hinojosa-Nogueira D., López-Maldonado A., Pastoriza S., Rufián-Henares J.Á. (2021). An in Vitro Batch Fermentation Protocol for Studying the Contribution of Food to Gut Microbiota Composition and Functionality. Nat. Protoc..

[67] Soltani S., Zirah S., Rebuffat S., Couture F., Boutin Y., Biron E., Subirade M., Fliss I. (2022). Gastrointestinal Stability and Cytotoxicity of Bacteriocins from Gram-Positive and Gram-Negative Bacteria: A Comparative in Vitro Study. Front. Microbiol..

[68] Naimi S., Zirah S., Hammami R., Fernandez B., Rebuffat S., Fliss I. (2018). Fate and Biological Activity of the Antimicrobial Lasso Peptide Microcin J25 under Gastrointestinal Tract Conditions. Front. Microbiol..

[69] Naimi S., Zirah S., Greppi A., Lacroix C., Rebuffat S., Fliss I. (2022). Impact of Microcin J25 on the Porcine Microbiome in a Continuous Culture Model. Front. Microbiol..

[70] Lopez F.E., Vincent P.A., Zenoff A.M., Salomón R.A., Farías R.N. (2007). Efficacy of Microcin J25 in Biomatrices and in a Mouse Model of *Salmonella* Infection. J. Antimicrob. Chemother..

[71] Said L.B., Emond-Rheault J.-G., Soltani S., Telhig S., Zirah S., Rebuffat S., Diarra M.S., Goodridge L., Levesque R.C., Fliss I. (2020). Phenomic and Genomic Approaches to Studying the Inhibition of Multiresistant Salmonella Enterica by Microcin J25. Environ. Microbiol..

[72] Telhig S., Pham N.P., Ben Said L., Rebuffat S., Ouellette M., Zirah S., Fliss I. (2024). Exploring the Genetic Basis of Natural Resistance to Microcins. Microb. Genom..

[73] Varas M.A., Muñoz-Montecinos C., Kallens V., Simon V., Allende M.L., Marcoleta A.E., Lagos R. (2020). Exploiting *Zebrafish Xenografts* for Testing the in Vivo Antitumorigenic Activity of Microcin E492 against Human Colorectal Cancer Cells. Front. Microbiol..

[74] Sassone-Corsi M., Nuccio S.-P., Liu H., Hernandez D., Vu C.T., Takahashi A.A., Edwards R.A., Raffatellu M. (2016). Microcins Mediate Competition among Enterobacteriaceae in the Inflamed Gut. Nature.

[75] Son Y., Kim B., Kim P., Min J., Park Y., Yang J., Kim W., Toyofuku M., Park W. (2024). Unexpected Vulnerability of Enterococcus Faecium to Polymyxin B under Anaerobic Condition. Gut Microbes.

[76] Kheadr E., Zihler A., Dabour N., Lacroix C., Blay G.L., Fliss I. (2010). Study of the Physicochemical and Biological Stability of Pediocin PA-1 in the Upper Gastrointestinal Tract Conditions Using a Dynamic in Vitro Model. J. Appl. Microbiol..

[77] Umu Ö.C.O., Bäuerl C., Oostindjer M., Pope P.B., Hernández P.E., Pérez-Martínez G., Diep D.B. (2016). The Potential of Class II Bacteriocins to Modify Gut Microbiota to Improve Host Health. PLoS ONE.

[78] Le Blay G., Lacroix C., Zihler A., Fliss I. (2007). In Vitro Inhibition Activity of Nisin a, Nisin Z, Pediocin PA-1 and Antibiotics against Common Intestinal Bacteria. Lett. Appl. Microbiol..

[79] Dabour N., Zihler A., Kheadr E., Lacroix C., Fliss I. (2009). In Vivo Study on the Effectiveness of Pediocin PA-1 and *Pediococcus acidilactici* Ul5 at Inhibiting *Listeria monocytogenes*. Int. J. Food Microbiol..

[80] Józefiak D., Kierończyk B., Juśkiewicz J., Zduńczyk Z., Rawski M., Długosz J., Sip A., Højberg O. (2013). Dietary Nisin Modulates the Gastrointestinal Microbial Ecology and Enhances Growth Performance of the Broiler Chickens. PLoS ONE.

[81] Kierończyk B., Rawski M., Mikołajczak Z., Świątkiewicz S., Józefiak D. (2020). Nisin as a Novel Feed Additive: The Effects on Gut Microbial Modulation and Activity, Histological Parameters, and Growth Performance of Broiler Chickens. Animals.

[82] Małaczewska J., Kaczorek-Łukowska E. (2021). Nisin—A Lantibiotic with Immunomodulatory Properties: A Review. Peptides.

[83] Gong J., Yu H., Liu T., Gill J.J., Chambers J.R., Wheatcroft R., Sabour P.M. (2008). Effects of Zinc Bacitracin, Bird Age and Access to Range on Bacterial Microbiota in the Ileum and Caeca of Broiler Chickens. J. Appl. Microbiol..

[84] Pedroso A.A., Menten J.F., Lambais M.R., Racanicci A.M.C., Longo F.A., Sorbara J.O.B. (2006). Intestinal Bacterial Community and Growth Performance of Chickens Fed Diets Containing Antibiotics. Poult. Sci..

[85] Xiong W., Wang Y., Sun Y., Ma L., Zeng Q., Jiang X., Li A., Zeng Z., Zhang T. (2018). Antibiotic-Mediated Changes in the Fecal Microbiome of Broiler Chickens Define the Incidence of Antibiotic Resistance Genes. Microbiome.

[86] Miles T.D., McLaughlin W., Brown P.D. (2006). Antimicrobial Resistance of *Escherichia coli* isolates from Broiler Chickens and Humans. BMC Vet. Res..

[87] Khong M.J., Snyder A.M., Magnaterra A.K., Young M.M., Barbieri N.L., Weimer S.L. (2023). Antimicrobial Resistance Profile of *Escherichia coli* Isolated from Poultry Litter. Poult. Sci..

[88] Barbosa A.A.T., de Melo M.R., da Silva C.M.R., Jain S., Dolabella S.S. (2021). Nisin Resistance in Gram-Positive Bacteria and Approaches to Circumvent Resistance for Successful Therapeutic Use. Crit. Rev. Microbiol..

[89] Solbiati J.O., Ciaccio M., Farías R.N., Salomón R.A. (1996). Genetic Analysis of Plasmid Determinants for Microcin J25 Production and Immunity. J. Bacteriol..

[90] Bennett S., Ben Said L., Lacasse P., Malouin F., Fliss I. (2021). Susceptibility to Nisin, Bactofencin, Pediocin and Reuterin of Multidrug Resistant *Staphylococcus aureus*, *Streptococcus dysgalactiae* and *Streptococcus uberis* Causing Bovine Mastitis. Antibiotics.

[91] Asare P.T., Greppi A., Pennacchia A., Brenig K., Geirnaert A., Schwab C., Stephan R., Lacroix C. (2021). In Vitro Modeling of Chicken Cecal Microbiota Ecology and Metabolism Using the PolyFermS Platform. Front. Microbiol..

[92] Hall M., Beiko R.G. (2018). 16s rRNA Gene Analysis with Qiime2. Methods Mol. Biol..

[93] Callahan B.J., McMurdie P.J., Rosen M.J., Han A.W., Johnson A.J.A., Holmes S.P. (2016). Dada2: High Resolution Sample Inference from Illumina Amplicon Data. Nat. Methods.

[94] Quast C., Pruesse E., Yilmaz P., Gerken J., Schweer T., Yarza P., Peplies J., Glöckner F.O. (2013). The Silva Ribosomal Rna Gene Database Project: Improved Data Processing and Web-Based Tools. Nucleic Acids Res..

[95] Wickham H. (2011). Ggplot2. WIREs Comput. Stat..

[96] R Core Team R: The R Project for Statistical Computing. https://www.r-project.org/.

