# Supplementary material for: In Vitro Insights into Bacteriocin-Mediated Modulation of Chicken Cecal Microbiota
Source: Int J Mol Sci. 2025 Jan 17;26(2):755. doi: 10.3390/ijms26020755 (PMC11765717; doi:10.3390/ijms26020755)
Supplement: Supplementary file 1 [file ijms-26-00755-s001.zip › ijms-3382146-supplementary.pdf]

In Vitro Insights into Bacteriocin-Mediated Modulation of Chicken Cecal Microbiota

Amal Mamjoud [1,2,3], Séverine Zirah [1,*], Eric Biron [3,4], Omar Fliss [2,3] and Ismail Fliss [2,3,*]

[1] Communication Molecules and Adaptation of Microorganisms (MCAM), Muséum National d'Histoire Naturelle, Centre National de la Recherche Scientifique, Paris, 75005, France; amal.mamjoud.1@ulaval.ca
[2] Food Science Department, Food and Agriculture Faculty, Université Laval, Quebec, QC G1V 0A6, Canada; omar.fliss.1@ulaval.ca
[3] Institute of Nutrition and Functional Foods, Université Laval, Quebec, QC, G1V 0A6, Canada;
eric.biron@pha.ulaval.ca
[4] Faculty of Pharmacy, Université Laval and Centre de Recherche du CHU de Québec-Université Laval,
Quebec, QC, G1V 0A6, Canada
* Correspondence: severine.zirah@mnhn.fr (S.Z.); ismail.fliss@fsaa.ulaval.ca (I.F.); Tel.: +33-01-4079-3140 (S.Z.); +1-418-656-2131 (ext. 406825) (I.F.)

SUPPLEMENTARY INFORMATION

Academic Editors: Carlo Genovese and Francesco Pegreffi

Received: 4 December 2024
Revised: 9 January 2025
Accepted: 14 January 2025
Published: date

Citation: Mamjoud, A.; Zirah, S.; Biron, E.; Fliss, O.; Fliss, I. In Vitro Insights into Bacteriocin-Mediated Modulation **of** Chicken Cecal Microbiota. *Int. J. Mol. Sci.* **2025**, *26*, x. https://doi.org/10.3390/xxxxx

Copyright: © 2025 by the authors. Submitted for possible open access publication under the terms and conditions of the Creative Commons Attribution (CC BY) license (https://creativecommons.org/licenses/by/4.0/).

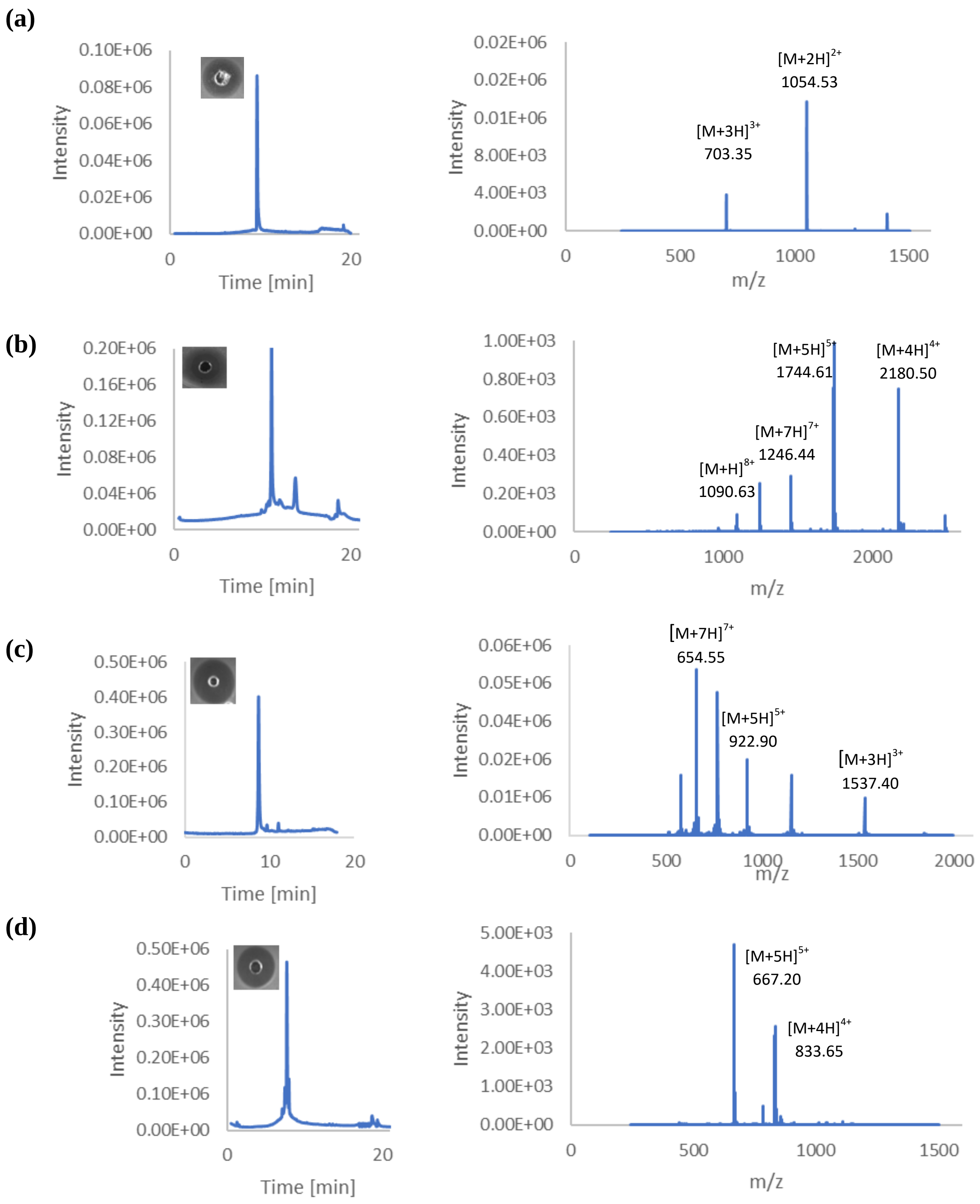

Figure S1. Bacteriocin purification quality control. Activity test by agar diffusion, LC profiles (total ion chromatograms, on the left) and MS spectra (on the right) of (a) microcin J25, (b) microcin E492, (c) pediocin PA-1 (M31L) and (d) nisin Z.

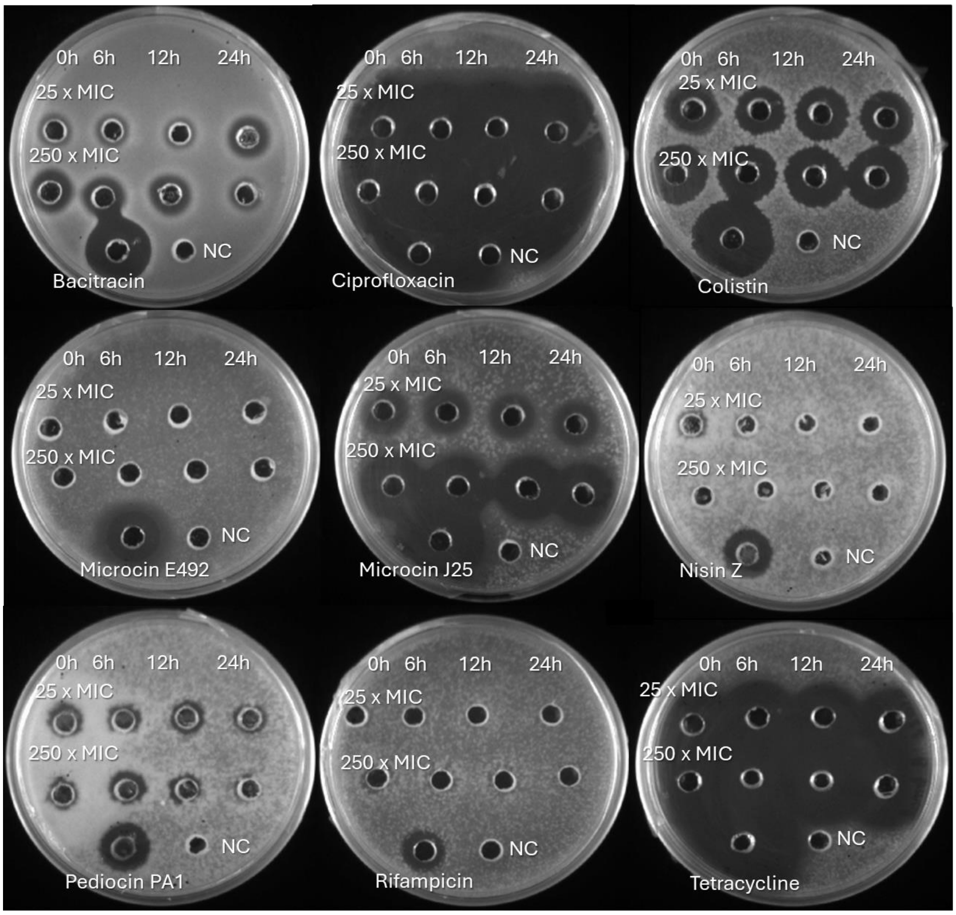

Figure S2. Agar diffusion tests of residual bacteriocin and antibiotic activity over time in the cecal fermentation supernatant, after introduction of 25 or 250 times the minimal inhibitory concentration (MIC). The indivative strains were *Streptococcus pyogenes RBL4,* for bacitracin, *Listeria ivanovii* HPB28 for nisin Z and pediocin PA-1 M31L, and *Salmonella enterica* Newport ATCC 6962 for all others. NC is the negative control, beside the positive control (compound freshly dissolved in buffer at 250 times the MIC). When present, inhibitory activity was stable over time in bacterial culture.

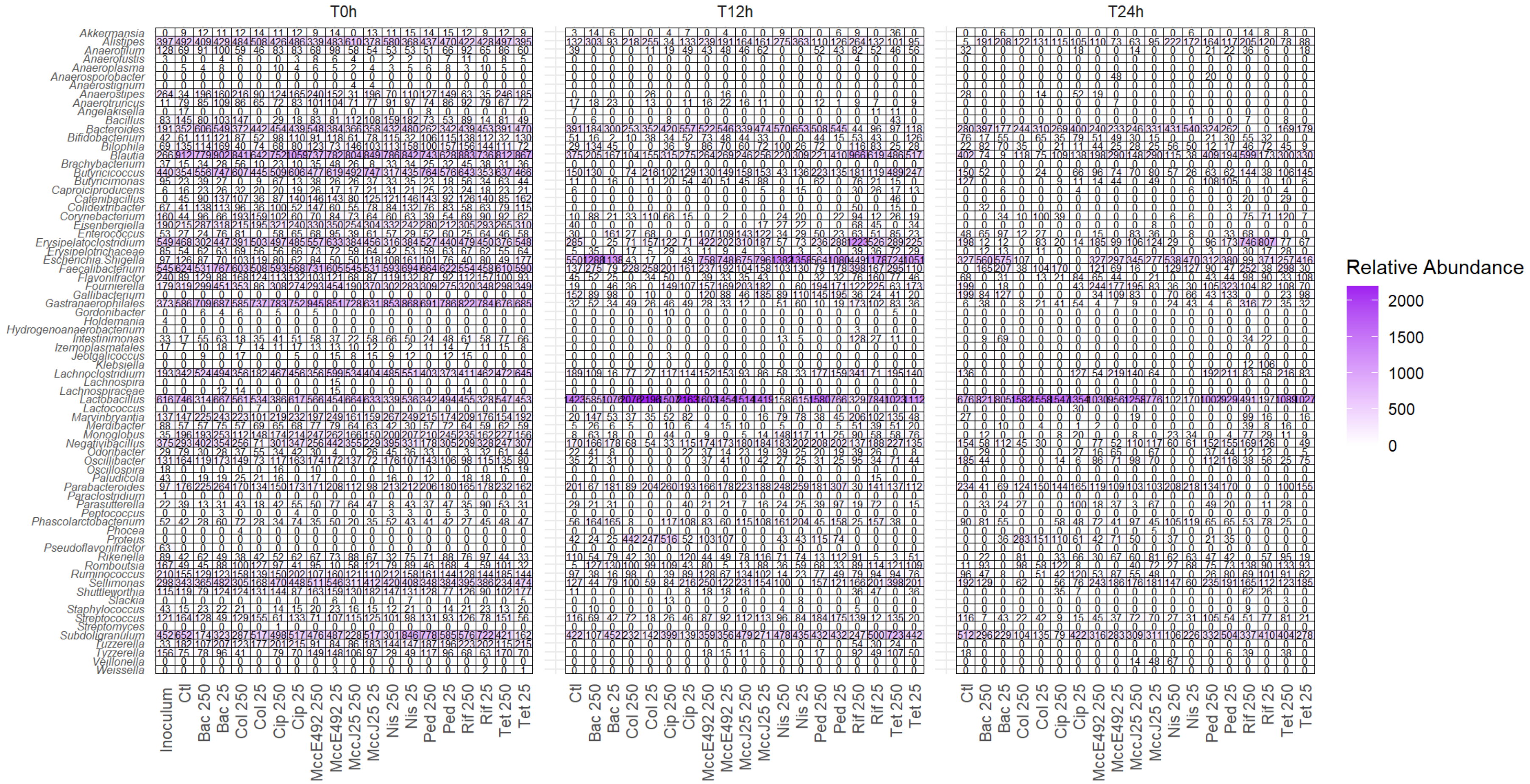

Figure S3. Bacterial diversity (genera) of poultry cecal microbiota in vitro (at 0 h, 12 h and 24 h). The relative abundance is represented on a numerical scale and a color intensity scale. Values are the average of three biological replicates for each fermentation. All ASV sequences shared in all treatments are represented. Bacitracin (Bac), colistin (Col), ciprofloxacin (Cip), microcin E492 (MccE492), microcin J25 (MccJ25), nisin Z (Nis), pediocin PA1 (M31L) (Ped), tetracycline (Tet) and rifampicin (Rif).
